# A Multi‐Stage Framework for Diagnosing Structural Redundancy Between Selected Functional Diversity Metrics: Empirical Applications Under an A Priori Expectation of Overlap

**DOI:** 10.1002/ece3.74671

**Published:** 2026-11-11

**Authors:** Gonzalo Sotomayor, Marie Anne Eurie Forio, Christine van der heyden, Patricio X. Lozano Rodríguez, Peter L. M. Goethals

**Affiliations:** ^1^ Facultad de Ingeniería Universidad de los Hemisferios, Paseo de la Universidad Quito Ecuador; ^2^ Faculty of Bioscience Engineering, Department of Animal Sciences and Aquatic Ecology Ghent University Ghent Belgium; ^3^ Faculty of Science and Technology – Health and Water Technology Research Center Hogeschool Gent/University of Applied Sciences and Arts HOGENT Ghent Belgium; ^4^ Facultad de Recursos Naturales, Museo de Entomología Escuela Superior Politécnica de Chimborazo (ESPOCH) Riobamba Ecuador

**Keywords:** benthic macroinvertebrates, diagnostic framework, functional diversity metrics, structural redundancy

## Abstract

1. Functional diversity (FD) is widely used to study ecosystem processes, leading to the continuous development of FD metrics. However, the proliferation of these descriptors raises concerns regarding structural redundancy, particularly in ecological modelling, where the simultaneous use of highly concordant FD metrics may compromise model parsimony.
2. We propose a multi‐stage approach to diagnose structural redundancy among selected FD metrics when theoretical, mathematical, or empirical considerations suggest overlap, using complementary analyses across continuous and categorised representations. We illustrate the approach through two pairwise comparisons: the community‐based weighted functional dendrogram index (wFDc), which incorporates the species community pool, versus richness–quadratic entropy (RQ) and Rao's quadratic entropy (Q) versus functional dispersion (FDis). The framework was applied to both metric pairs using benthic macroinvertebrate assemblages from three contrasting freshwater ecosystems in Ecuador.
3. Both metric pairs exhibited high concordance across the three ecosystems, with strong continuous associations and substantial agreement in percentile‐based classifications. By incorporating richness‐controlled analyses, the framework evaluates whether concordance persists after accounting for shared richness dependence. For both pairs, residual associations remained strong, supporting suspected structural overlap in the examined datasets.
4. Our findings suggest that the simultaneous use of highly concordant FD metrics could be unnecessary. Rather than questioning the development of new FD metrics, the proposed framework provides a practical diagnostic protocol for evaluating their structural redundancy. To facilitate its application, we provide the R package FD Concordance https://github.com/Gonpa83/FD_concordance.git and a companion Shiny application of the same name https://01a168d6‐1a69‐a0e6‐7a29‐7e329e9dbbdb.share.connect.posit.cloud/ enabling researchers to apply the protocol to their own index values. These tools support targeted assessments of suspected overlap and more parsimonious ecological modelling and biomonitoring.

## Graphical abstract

We present a multi‐stage framework for evaluating structural redundancy among selected functional diversity metrics when prior evidence suggests overlap. Comparisons of wFDc–RQ and Q–FDis across three freshwater ecosystems in Ecuador revealed strong concordance that persisted after accounting for taxonomic richness. The FD Concordance R package and companion Shiny application enable researchers to apply the protocol to their own index values, supporting metric selection for ecological modelling and biomonitoring.

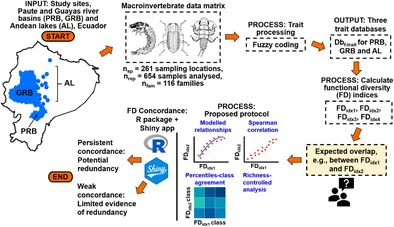

## 1 Introduction

Biodiversity is described through multiple dimensions, including genetic, taxonomic and community‐level variation (Díaz and Malhi 2022). Numerous indices capture these facets of biological structure (Sîrbu et al. 2025) and are applied across terrestrial, marine and freshwater ecosystems from both taxonomic and functional perspectives (Shah Esmaeili et al. 2022). In freshwater systems, ecological assessments have prioritised taxonomic approaches, with benthic macroinvertebrates serving as the most used bioindicators (Heino et al. 2022). Recently, however, ecological studies have increasingly adopted functional diversity (FD) indices, as trait‐based approaches can provide deeper insights into ecosystem functioning and improve biomonitoring effectiveness (Kaushik et al. 2025). While trait‐based approaches provide complementary ecological information beyond species‐based models (Zakharova et al. 2019), the increasing number of FD metrics has created a need for systematic frameworks to evaluate their relationships and potential redundancy. FD indices are frequently assumed to provide complementary information, yet this assumption is rarely tested, particularly when related mathematical foundations suggest potential overlap (Maire et al. 2015). When FD indices capture overlapping aspects of functional structure, they introduce unnecessary analytical complexity and may bias ecological interpretations (Guillerme et al. 2025; Mammola et al. 2021). The robustness of these metrics to common biases (such as species richness effects and data structure constraints) remains insufficiently evaluated in empirical contexts (Daly et al. 2018). There is a need for a robust framework to evaluate whether different FD indices converge on similar representations of functional information and how such convergence is influenced by the choice of analytical approach (e.g., gradient‐based versus discretized representations). To evaluate potential structural redundancy between selected FD metrics for which theoretical, mathematical, or empirical considerations suggest overlap, we propose a multi‐stage analytical framework integrating complementary statistical approaches. We illustrate its application through two pairwise comparisons. First, we examine the recently proposed richness–quadratic entropy index (RQ; Pavoine 2026a), defined as the product of taxonomic richness (R) and Rao's quadratic entropy (Q; Rao 1982), alongside the established community‐based weighted functional dendrogram index incorporating the species community pool (wFDc; Casanoves et al. 2011). We hypothesise that these indices may exhibit structural concordance. Although they differ in mathematical construction, both summarise community‐level functional structure, providing an a priori motivation to investigate potential overlap rather than assume their independence. Second, we compare Rao's quadratic entropy with functional dispersion (FDis). Their close relationship was already described by Laliberté and Legendre (2010), providing a literature‐based rationale for applying the protocol. The framework integrates complementary analyses of continuous and discretized ecological variation to evaluate concordance (agreement between metric outputs), redundancy (overlapping functional information), and potential collinearity (statistical dependence arising when concordant metrics are jointly incorporated into ecological models) between selected FD metrics while explicitly accounting for known sources of bias, such as taxonomic richness. Using benthic macroinvertebrate assemblages as a model community, we demonstrate the framework through two pairwise comparisons of FD metrics: RQ versus wFDc and Q versus FDis. The framework provides a diagnostic protocol for targeted comparisons between selected FD metrics when an a priori rationale exists for investigating potential structural overlap.

## 2 Materials and Methods

### 2.1 Study Area

The study encompassed three freshwater ecosystems in Ecuador: the Paute and Guayas river basins, and 34 lakes distributed across the north‐central Andes (Figure 1). The Paute River basin covers 6442 km^2^ and ranges in elevation from ~410 to 4680 m above sea level (m a.s.l.), with mountainous topography shaped by glacial processes. Mean annual discharge is ~136.3 m^3^ s^−1^, supporting hydropower generation. The Guayas River basin covers 33,700 km^2^ and spans elevations from 0 to 5063 m a.s.l., including coastal lowlands, foothills and Andean headwaters. Mean annual discharge is ~970 m^3^ s^−1^. The basin supports agricultural, urban and hydropower activities. The lakes span elevations from 2197 to 4337 m a.s.l. and surface areas from 0.17 to 541.21 ha. Together, these systems represent a broad environmental gradient for evaluating FD metric redundancy across contrasting ecological conditions.

**FIGURE 1:**
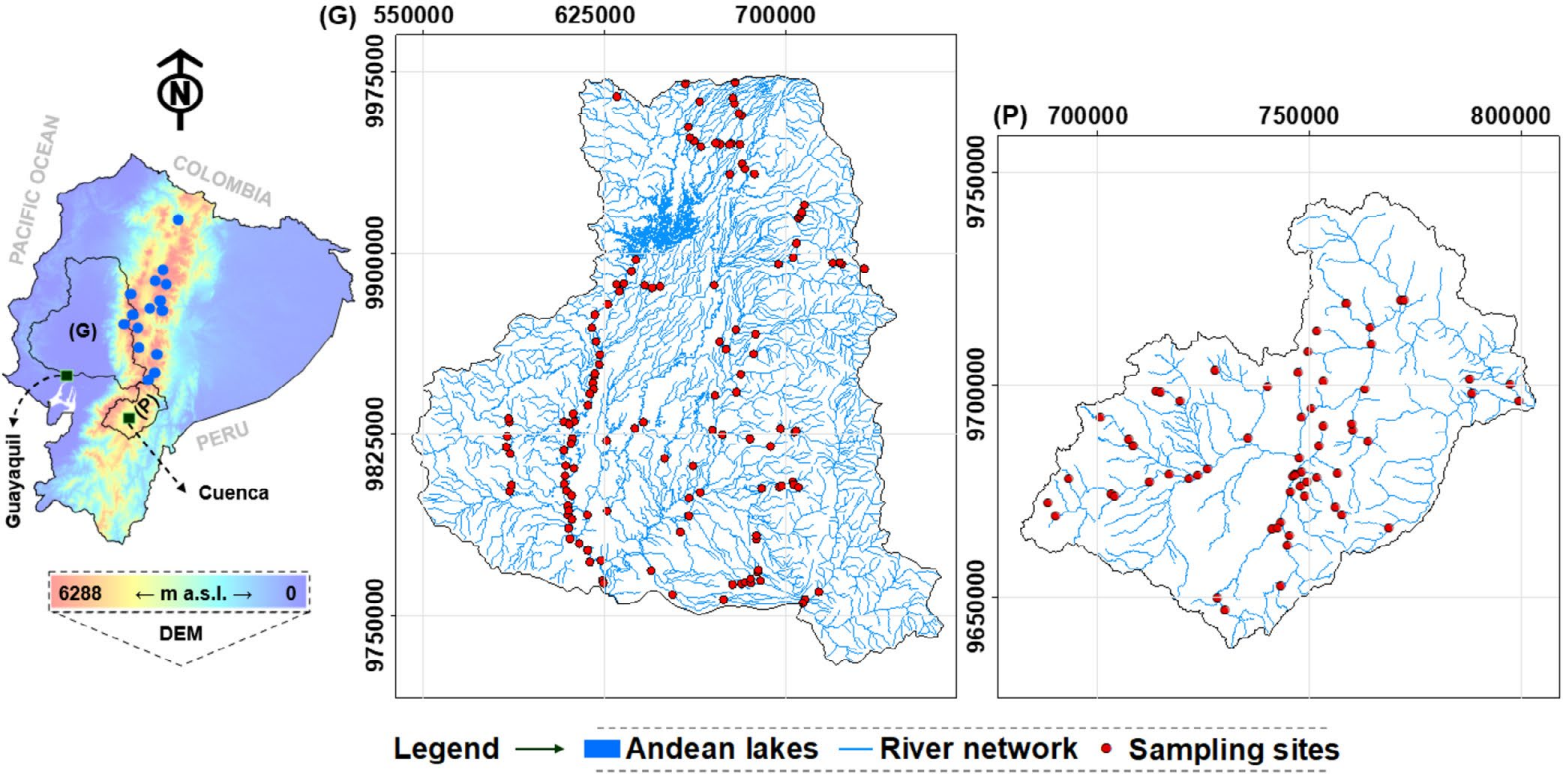
Left panel: Location of the Guayas (G) and Paute (P) basins in continental Ecuador overlaid on a digital elevation model (DEM). Elevation is expressed in metres above sea level (m a.s.l.). Blue points indicate the locations of high‐altitude lake systems in the Andes. For national‐scale visualisation, lake symbols were slightly enlarged to improve readability. Panels G and P show the river network and sampling locations in the Guayas and Paute basins, respectively. Coordinate system: WGS84 UTM 17S (m).

### 2.2 Sampling Design

For the Paute River basin, benthic macroinvertebrate data comprised 390 sampling observations from 64 sites monitored in 2008, 2010–2013 and 2017. Sites were revisited multiple times, with impacted and reference sites sampled more frequently. Monitoring effort was lower in 2008 and 2017. Each sampling event constituted one observation. For the Guayas River basin, 163 sites were surveyed once. The Andean lakes dataset comprised 101 observations from 34 lakes sampled in 2021, with up to three sampling sites per lake.

### 2.3 Benthic Macroinvertebrate Sampling

In the Paute and Guayas river basins, benthic macroinvertebrates were collected by kick‐sampling using a 500 μm mesh hand net. In the Guayas River basin, sampling covered 10–20 m reaches, with effort distributed proportionally across microhabitats. In the Paute River basin, each 20 m reach was subdivided into three transects and sampled for 3 min per transect, encompassing variation in substrate, depth and flow velocity. In Andean lakes, a D‐frame hand net (500 μm mesh) was used along three independent 40 m shoreline transects per lake. Sampling effort was distributed proportionally across aquatic habitats, with a fixed duration of 5 min per transect. Surveyed microhabitats included benthic substrates (stones, sand and mud), macrophyte zones (floating, submerged and emergent vegetation), and other submerged structures. In all cases, hand‐net sampling was complemented by manual collection with forceps when necessary. Samples were preserved in ethanol and identified to the family level.

### 2.4 Functional Trait Database

To characterise the functional structure of macroinvertebrate communities, we selected eight biological traits comprising 42 functional categories (Table 1), following established frameworks for Neotropical taxa (Paz et al. 2023). A family‐level trait database (Db_f‐trait_) was compiled using affinity scores from Sotomayor et al. (2021) following the fuzzy coding approach (Chevene et al. 1994). Taxon affinities to functional categories were originally scored from 0 (no affinity) to 3 (high affinity).

**TABLE 1:** Functional traits and categories used in this study.

Trait	Category
Feeding habits	Collector‐Filterer
	Collector‐Gatherer
	Piercers
	Predators
	Scrapers
	Shredders
	Parasite
Respiration	Tegument
	Gill
	Plastron
	Spiracle
Body form	Streamlined
	Flattened
	Cylindrical
	Spherical
Maximum body size (mm)	< 2.5
	2.5–5.0
	5–10
	10–20
	20–40
	40–80
	> 80
Body flexibility	None (< 10°)
	Low (10°–45°)
	High (> 45°)
Locomotion	Flier
	Surface swimmer
	Full water swimmer
	Crawler
	Burrower
	Temporarily attached
Reproduction	Asexual
	Clutches & cemented
	Clutches & free
	Clutches in vegetation
	Clutches & Terrestrial
	Isolated eggs & clutches
	Isolated eggs & free
	Ovoviviparity
Exoskeleton hardness	None
	Moderate
	High

### 2.5 Functional Diversity Metrics

Using the corresponding Db_f‐trait_ matrices, four FD indices were computed for each sampling unit and evaluated through two pairwise comparisons, wFDc versus RQ and Q versus FDis. The wFDc index estimates FD as the total branch length of a community‐based functional dendrogram incorporating the community pool of taxa. Functional dissimilarities are weighted by the relative abundances of distinct functional trait profiles as $c_{\mathrm{ij}}^{\prime }=m\;c_{\mathrm{ij}}\;\sqrt{w_{i}\;w_{j}}$. Here, $c_{\mathrm{ij}}$ is the cophenetic distance between distinct functional trait profiles i and j, obtained from an average‐linkage dendrogram constructed from Euclidean distances across the community pool. The weights $w_{i}$ and $w_{j}$ are their relative abundances within the sampling unit, aggregated for taxa with identical trait profiles, and m is the number of distinct trait profiles present in that unit. The resulting weighted dissimilarity matrix is then used to construct the sampling‐unit dendrogram using Ward's method, whose total branch length gives wFDc (Casanoves et al. 2011). The RQ index (Pavoine 2026a) is defined as the product of taxonomic richness (R) and Rao's quadratic entropy (Q; Rao 1982), i.e., RQ = *R* × Q. Q was calculated as $Q=\frac{1}{2}\;\Sigma_{i}\Sigma_{j}\;\left(w_{i}\;w_{j}\;d_{\mathrm{ij}}^{2}\right)$, where $w_{i}$ and $w_{j}$ are the relative abundances of taxa and $d_{\mathrm{ij}}$ is their original Euclidean distance in functional trait space. Both sums extend over all taxa present in the sampling unit. Thus, wFDc represents FD through weighted dendrogram topology, whereas RQ combines pairwise functional dissimilarity with R. Although Gower‐based approaches are commonly used for functional trait data (Pavoine et al. 2009), Euclidean distance combined with Ward linkage has been previously applied for dendrogram‐based FD metrics, including wFDc (Roa‐Fuentes et al. 2022), providing a methodological precedent for the distance choice adopted here. To maintain methodological consistency and avoid introducing differences attributable to the choice of dissimilarity measure, Rao's quadratic entropy (Q) was computed from the same original Euclidean distance matrix and multiplied by richness to obtain RQ. For the second comparison, Q was evaluated as a standalone metric, without multiplication by richness, alongside FDis (Laliberté and Legendre 2010). FDis was calculated as the abundance‐weighted mean Euclidean distance of taxa to their abundance‐weighted community centroid in functional trait space. Q and FDis were calculated using the same trait information and relative abundances within each sampling unit. Within each pair, using the same trait information and distance basis reduced differences attributable to trait representation, allowing the comparison to focus on the indices' mathematical construction. Analyses were performed at the family level, consistent with established stream bioassessment protocols (Sotomayor et al. 2021).

### 2.6 Multi‐Stage Framework for Evaluating Functional Diversity Indices

To evaluate metric independence, we implemented a multi‐stage analytical framework. The approach is demonstrated through two pairwise comparisons, wFDc versus RQ and Q versus FDis, and is intended as a transferable diagnostic framework for evaluating selected FD metrics when prior evidence suggests potential structural overlap.

#### 2.6.1 Continuous Relationships: Polynomial and Additive Modelling

To assess the relationship between indices in continuous space, regression models were fitted using wFDc as the response variable and RQ as the predictor for the first comparison, and FDis as the response variable and Q as the predictor for the second. A second‐order polynomial regression was applied to the Guayas River basin, allowing detection of non‐linear relationships and potential saturation effects within each metric pair. Such patterns are consistent with previous FD studies, where increases in R do not translate into expansion of functional space (Schleuter et al. 2010). For datasets with longitudinal structures (Paute River basin and Andean lakes), Generalised Additive Mixed Models (GAMMs) were fitted to account for repeated observations from the same sampling units. Random intercepts for site or lake identity accounted for clustering within sampling units (Zuur et al. 2009). GAMMs incorporate smooth functions of predictor variables, enabling complex relationships to be modelled without specifying a predefined functional form (Wood 2017). GAMMs used a Gaussian distribution with an identity link and thin‐plate regression splines (*k* = 5). Additive mixed models were fitted using restricted maximum likelihood, whereas polynomial models were fitted using ordinary least squares. Model fit was assessed using R^2^ for polynomial models and adjusted R^2^ for GAMMs. Statistical significance was evaluated using overall model F‐tests for polynomial regressions and predictor smooth‐term tests for GAMMs.

#### 2.6.2 Monotonic Association: Spearman Correlation

To complement the regression analysis, Spearman rank correlation (r_s_) was calculated between wFDc and RQ and between Q and FDis. Coefficients were interpreted descriptively. This non‐parametric measure was selected because it does not assume linearity and is robust to non‐normal distributions and outliers. By evaluating whether both indices preserve the relative ordering of sampling units, r_s_ provides a complementary assessment of rank concordance, which is relevant for bioassessment applications where site prioritisation depends on consistent ordering of ecological quality (Zar 2014).

#### 2.6.3 Distribution‐Based Agreement: Percentile Classification

To evaluate agreement across comparable categories, the four indices were discretised into three classes (low, medium and high) using the 33rd and 67th percentiles (Sotomayor et al. 2020). This data‐driven approach aims to produce approximately balanced class sizes, reducing bias from uneven distributions and avoiding arbitrary thresholds. Agreement was quantified separately for each metric pair as global agreement (the proportion of matching classifications across observations) and balanced agreement (the mean class‐level Jaccard agreement) (Podani 2021). Class‐level Jaccard agreement was calculated as the ratio of the intersection to the union of observations assigned to each class by both indices. Despite the expected information loss associated with discretisation (Altman and Royston 2006), this analysis was included to evaluate whether both indices within each pair produce consistent classifications under decision‐oriented conditions, particularly in freshwater bioassessment where management decisions often rely on categorical quality thresholds.

#### 2.6.4 Residual Analysis Controlling for Taxonomic Richness

To evaluate whether the association between FD indices was driven by their shared dependence on taxonomic richness (R), a residual‐based analysis was performed for each metric pair. Richness‐related effects may inflate associations between indices and obscure whether they capture distinct aspects of functional structure; therefore, richness control was applied to both comparisons, irrespective of whether richness was explicitly incorporated into the index formula. Following the dataset‐specific modelling strategy described in Section 2.6.1, second‐order polynomial regressions were fitted for the Guayas dataset, whereas GAMMs were fitted for the Paute River and Andean lakes datasets to account for repeated observations within sites and lakes, respectively. For each dataset, the four indices were modelled separately as functions of R, and residuals were extracted from the corresponding models. Residuals were then correlated using Spearman's rank correlation coefficient (r_s_) within each pair, wFDc versus RQ and Q versus FDis. Residual correlation coefficients were interpreted descriptively. By removing the variation explained by richness while accounting for the underlying sampling structure, this approach isolates the component of each index not explained by the fitted richness models, providing a measure of structural association beyond R effects. A strong residual correlation indicates that both indices capture similar functional patterns independently of R, whereas a weak correlation suggests that their association is primarily driven by shared dependence on R (Legendre and Legendre 2012). Structural redundancy is supported when concordance is consistently strong across complementary analytical stages and persists after controlling for richness; conversely, weak or inconsistent concordance across analyses, or a marked loss of association after richness control, would indicate limited evidence of structural redundancy. The analytical workflow is summarised in Figure 2.

**FIGURE 2:**
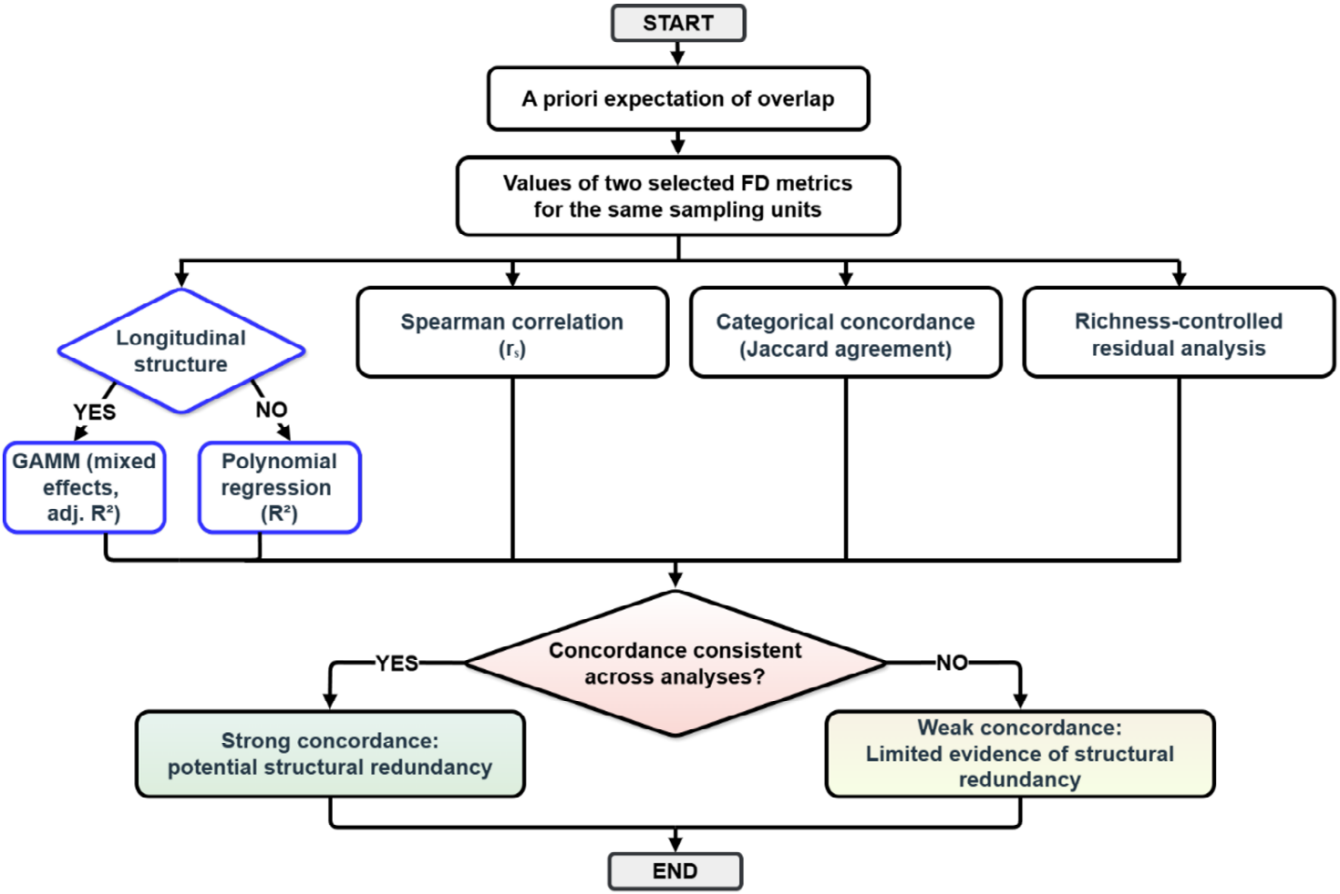
Flowchart of the multi‐stage framework for assessing potential structural redundancy between two selected functional diversity metrics under an a priori expectation of overlap. GAMM = Generalised Additive Mixed Model; R^2^ = coefficient of determination; adj. R^2^ = adjusted coefficient of determination; r_s_ = Spearman rank correlation coefficient.

We implemented the framework in FD Concordance, an R package and companion Shiny application, enabling researchers to evaluate suspected overlap between selected FD metrics using their own index values.

#### 2.6.5 Code and Software

Functional diversity indices were calculated using a custom R implementation based on FDiversity routines (Casanoves et al. 2011), reproducing the software's reference outputs in the validation checks performed. All subsequent analyses were performed in R‐software (v. 4.4.2; R Core Team 2024). Data preprocessing and visualisations were performed using the dplyr and ggplot2 packages, respectively (Wickham et al. 2014, 2026). Polynomial regression models were fitted using base R‐software, whereas GAMMs were fitted using the mgcv package (Wood 2000). Spearman correlations were computed using base R‐software. Data import and export used the readxl (Wickham and Bryan 2025) and writexl (Ooms 2025) packages, respectively. OpenAI's ChatGPT and Codex were used to assist with the development, debugging and testing of the FD Concordance package, and its companion Shiny application. The authors retain responsibility for the scientific content and software.

## 3 Results

### 3.1 Continuous Relationships: Polynomial and Additive Mixed Modelling

Regression analyses revealed a strong relationship between wFDc and RQ across all freshwater ecosystems (Figure 3a–c), with high explanatory power (R^2^ = 0.95 for the polynomial model and adj. R^2^ = 0.92–0.96 for the GAMMs; *p* < 0.001 in all cases). In the Guayas dataset (Figure 3a), the second‐order polynomial model identified a non‐linear relationship, with wFDc approaching an asymptote at higher RQ values. In the Paute dataset (Figure 3b), the GAMM showed a strong positive, non‐linear relationship. In contrast, the Andean Lakes dataset (Figure 3c) exhibited a near‐linear relationship, with the GAMM closely approximating a straight line. Despite these differences in functional form, observations remained tightly clustered around the fitted models (Figure 3a–c). Strong relationships were also observed between Q and FDis across all three ecosystems (Figure 3d–f), with slightly higher explanatory power than for wFDc versus RQ in each dataset (R^2^ = 0.98 for Guayas; adj. R^2^ = 0.98 for Paute and 0.99 for the Andean lakes; *p* < 0.001 in all cases). FDis increased non‐linearly with Q, with a progressively declining slope at higher Q values. Observations were tightly clustered around the fitted curves across the three datasets. High R^2^ (and adj. R^2^) values across contrasting model structures and geographic regions support structural concordance within both metric pairs.

**FIGURE 3:**
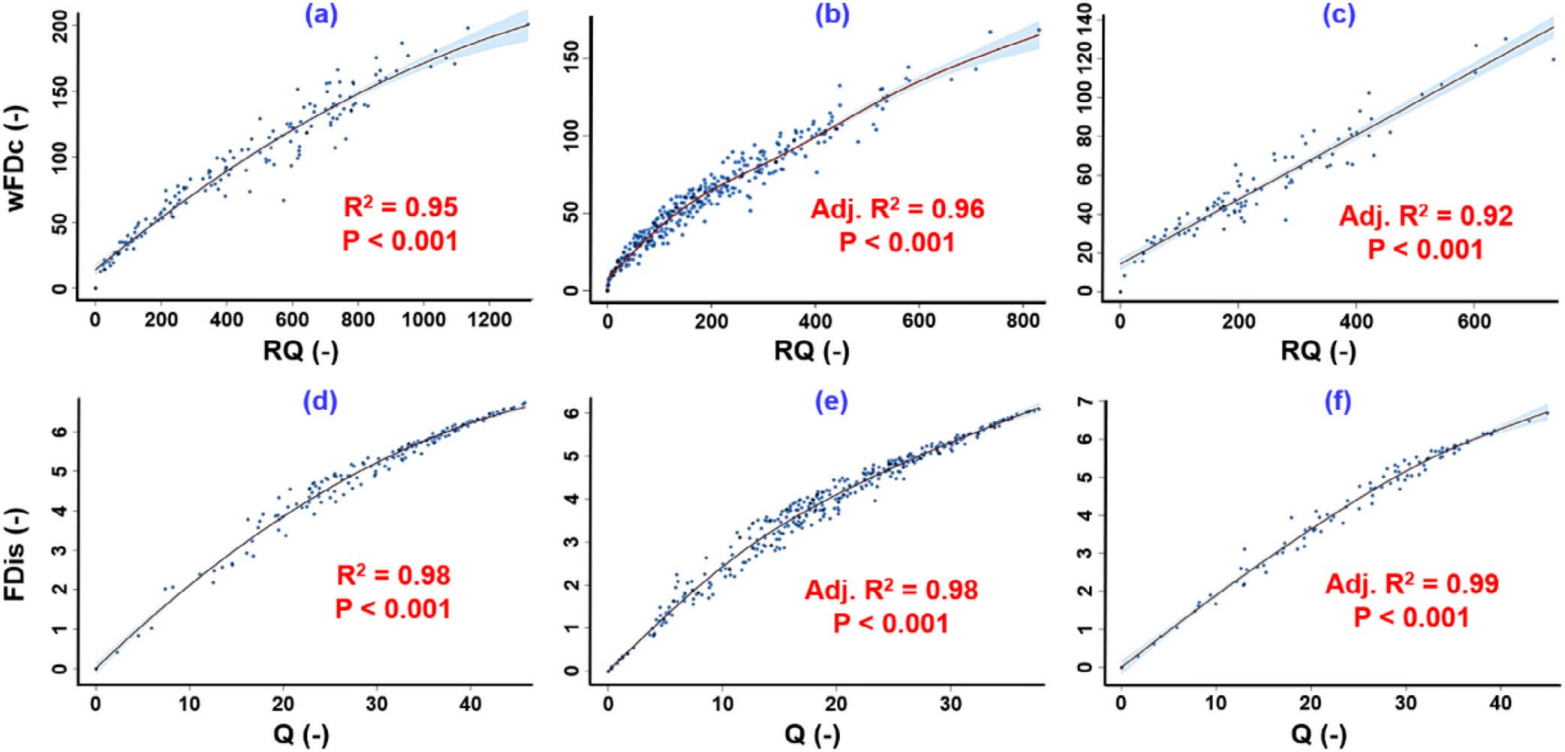
Relationships between wFDc and RQ in panels (a–c), and between Q and FDis in panels (d–f). Columns represent the Guayas River basin (a, d), Paute River basin (b, e), and Andean lakes (c, f). Fitted lines represent second‐order polynomial models for Guayas and Generalised Additive Mixed Models (GAMMs) for Paute and the Andean lakes. Shaded bands indicate approximate pointwise 95% confidence intervals for the fitted mean. GAMM curves exclude random intercepts. FDis = functional dispersion; Q = Rao's quadratic entropy; *R* = richness; R^2^ = coefficient of determination, adj; R^2^ = adjusted coefficient of determination; wFDc = functional diversity based on dendrograms including the community pool;.

### 3.2 Monotonic Association: Spearman Correlation

Spearman rank correlation confirmed a strong association between wFDc and RQ across datasets. Correlation coefficients were consistently high in the Guayas (r_s_ = 0.97) and Paute (r_s_ = 0.98) river basins, and the Andean lakes (r_s_ = 0.93), indicating monotonic agreement between the indices. Q and FDis also exhibited strong monotonic associations, with slightly higher correlation coefficients in all three datasets (r_s_ = 0.99 for Guayas, Paute and the Andean lakes). These results reinforce the regression analyses, demonstrating that the association is maintained regardless of the functional form of the relationship.

### 3.3 Distribution‐Based Agreement: Percentile Classification

Discretisation of wFDc and RQ revealed high categorical agreement across datasets (Table 2; Figure 4a). Global agreement reached 0.87 in the Guayas River basin, 0.90 in the Paute River basin and 0.77 in the Andean lakes. Balanced agreement, calculated as the mean class‐level Jaccard agreement, was lower in the Andean lakes (0.63) than in the Guayas (0.77) and Paute (0.82) river basins. Class‐level agreement varied among datasets (Figure 4a). The highest Jaccard agreement occurred for the low class in Guayas (0.90) and Paute (0.87), whereas the high class showed the highest agreement in the Andean lakes (0.74). The medium class showed the lowest agreement in all datasets. Q and FDis exhibited higher global and balanced agreement than wFDc and RQ in each dataset (Table 2; Figure 4b). Global agreement reached 0.91 in Guayas and 0.90 in both Paute and the Andean lakes. Balanced agreement reached 0.84, 0.82 and 0.82, respectively. Class‐level Jaccard agreement was highest for the high class in Guayas (0.93) and Paute (0.91), and for the low class in the Andean lakes (0.89). As observed for wFDc and RQ, the medium class showed the lowest agreement in all three datasets (0.74–0.77).

**TABLE 2:** Global and balanced agreement based on percentile classification for two pairs of functional diversity metrics across the three freshwater ecosystems: WFDc versus RQ and Q versus FDis.

Freshwater ecosystem	wFDc–RQ: global agreement	wFDc–RQ: balanced agreement	Q–FDis: global agreement	Q–FDis: balanced agreement
Guayas River basin	0.87	0.77	0.91	0.84
Paute River basin	0.90	0.82	0.90	0.82
Andean lakes	0.77	0.63	0.90	0.82

Abbreviations: FDis = functional dispersion; Q = Rao's quadratic entropy; *R* = richness; RQ = richness–quadratic entropy; wFDc = functional diversity based on dendrograms including the community pool.

**FIGURE 4:**
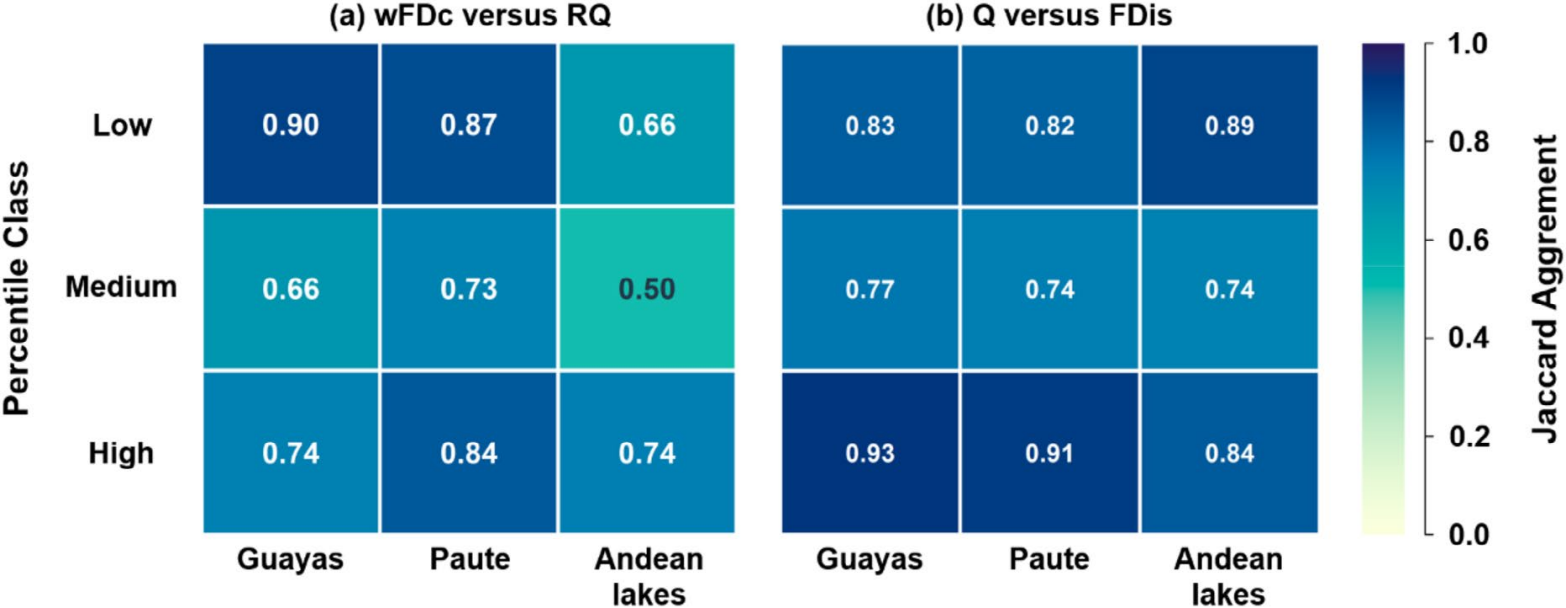
Class‐level Jaccard agreement between (a) wFDc and RQ and (b) Q and FDis in the Guayas and Paute river basins and the Andean lakes. Indices were grouped into percentile‐based classes. Cell values indicate Jaccard agreement rounded to two decimal places; both panels share a colour scale from 0 to 1. FDis = functional dispersion; Q = Rao's quadratic entropy; RQ = Q × taxonomic richness; wFDc = functional diversity based on dendrograms including the community pool.

### 3.4 Residual Analysis Controlling for Taxonomic Richness

Residual analysis confirmed a robust association between wFDc and RQ after controlling for taxonomic richness (R) (Figure 5). Spearman correlation coefficients between residuals remained high in the Guayas (r_s_ = 0.79), Paute (r_s_ = 0.89) and Andean lakes (r_s_ = 0.84) datasets. These results demonstrate that concordance between wFDc and RQ is not merely an artefact of their shared dependence on R, but reflects structural overlap between the two functional descriptors. Residual scatterplots (Figure 5) showed a clear monotonic pattern across all ecological contexts, particularly in the Paute dataset, indicating substantial overlap in the functional information captured by both indices after accounting for richness. Strong residual associations were also observed between Q and FDis across all three ecosystems (Figure 5). Spearman correlation coefficients reached 0.97 in Guayas, 0.97 in Paute and 0.99 in the Andean lakes, exceeding the corresponding coefficients for wFDc and RQ. These results indicate that the strong concordance between Q and FDis persisted after accounting for richness, supporting potential structural overlap in the examined datasets.

**FIGURE 5:**
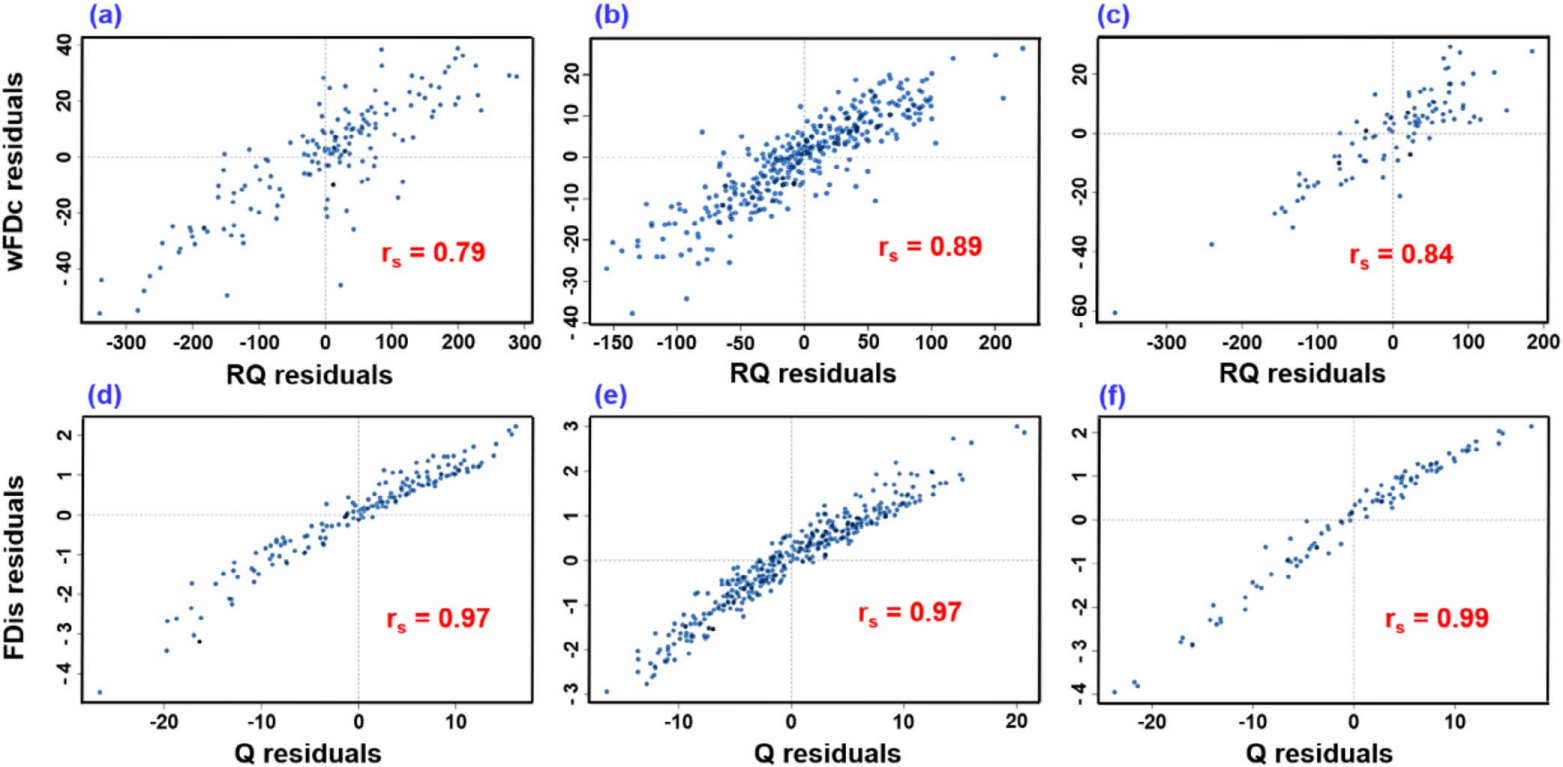
Relationships between richness‐controlled residuals of wFDc and RQ in panels (a–c), and of Q and FDis in panels (d–f). Columns represent the Guayas River basin (a, d), Paute River basin (b, e) and Andean lakes (c, f). Dotted lines indicate zero residual values. FDis = functional dispersion; Q = Rao's quadratic entropy; *R* = richness; RQ = richness–quadratic entropy; r_s_ = Spearman rank correlation coefficient; wFDc = functional diversity based on dendrograms including the community pool.

## 4 Discussion

The proliferation of functional diversity (FD) indices reflects a growing capacity to capture nuanced facets of ecosystem functioning. However, as the ecological toolbox expands, it remains unclear whether newly proposed metrics provide genuinely independent information or contribute to redundancy (Guillerme et al. 2025). While previous research has extensively examined the congruence between taxonomic and functional indices (Paz et al. 2023; Bandeira et al. 2013), the structural overlap among FD metrics remains under‐explored. Using benthic macroinvertebrate communities from three contrasting freshwater ecosystems in Ecuador as a case study, we introduce a transferable framework for evaluating structural redundancy between selected FD metrics when theoretical, mathematical, or empirical considerations provide an a priori rationale for expecting overlap. Rather than questioning the development of new indices, the framework provides a structured diagnostic approach to assess their added value, identify potential redundancy and improve the interpretability of functional assessments.

### 4.1 Multi‐Step Concordance of Functional Structure

The implemented approach revealed a consistent pattern across the complementary analytical steps used to compare the two pairs of FD indices, wFDc versus RQ and Q versus FDis. Regression models showed strong associations within each pair across the evaluated freshwater ecosystems, further supported by high Spearman correlation coefficients and substantial categorical agreement based on percentile‐derived classes. Concordance was strongest when the indices within each pair were evaluated along their full numerical gradients, whereas the discretised representation provided a more conservative summary. This variation in agreement is statistically expected; the conversion of continuous variables into discrete categories leads to information loss and reduced statistical sensitivity (MacCallum et al. 2002). By collapsing fine‐scale differences into a few classes, the resolution of the functional structure is reduced, attenuating the observed strength of concordance between indices. However, this reduced sensitivity should not be interpreted as a limitation of the categorical approach. In freshwater assessment, categorisations (e.g., water quality classes 1, 2, or 3) are often essential because they are embedded within regulatory frameworks and used for management communication (Hering et al. 2010).

### 4.2 Functional Congruence and Dimensional Overlap Between Indices

Motivated by the elegant description of the RQ index (Pavoine 2026a), our results reveal congruent structures for RQ and the established wFDc index (Casanoves et al. 2011). Despite their distinct statistical foundations—wFDc is based on functional dendrograms (Petchey and Gaston 2007), whereas RQ originates from the product of Rao's Quadratic Entropy (Q) (Rao 1982) and taxonomic richness (R)—both indices appear to capture overlapping components of the same functional structure. The likely explanation lies in the underlying topology of the functional trait space. While wFDc relies on branching length and RQ reflects expected pairwise dissimilarities, both are driven by the distribution of functional trait categories within the community. Both indices can therefore be interpreted as alternative projections of the same functional space, quantifying the overall dispersion of taxa within trait space. The Q–FDis comparison extends this application to a second pair whose close relationship has already been documented (Laliberté and Legendre 2010). Both metrics describe aspects of abundance‐weighted dispersion in functional trait space, although Q is based on pairwise distances and FDis on distances to an abundance‐weighted centroid. Their strong concordance across the three ecosystems, including after richness control, supports potential overlap in the information they provide in these datasets. This comparison illustrates how the framework can examine a previously documented relationship alongside the original RQ–wFDc hypothesis, without treating concordance as evidence of universal equivalence. We do not claim that this concordance diminishes the relevance of any of the evaluated indices. Rather, our results suggest that, when there is an a priori rationale for expecting structural overlap and the aim is not to compare metric performance (e.g., Botta‐Dukát and Czúcz 2016), the simultaneous use of indices capturing the same functional structure may be unnecessary. Rather than promoting comparisons in the absence of such a rationale, the proposed framework provides an approach for testing whether suspected redundancy is supported by empirical evidence. In ecological modelling, where predictor redundancy can obscure interpretation, increase model complexity and reduce inferential efficiency (Galland et al. 2020), a parsimonious selection of metrics is recommended, prioritising those that most efficiently capture the dominant functional structure.

### 4.3 Richness Effects and Functional Saturation

A central challenge in comparing FD indices lies in disentangling structural concordance from associations driven by shared dependence on taxonomic richness (R). Although not all FD indices incorporate R explicitly, most are inherently influenced by it, as the number of taxa constrains the configuration and occupancy of the functional trait space (Pavoine 2026b). From an ecological perspective, FD is often assumed to exhibit an asymptotic relationship with R: as more taxa are added to a community, the probability increases that new species contribute to already represented functional groups rather than introducing entirely new functions (Mouillot et al. 2011; Petchey and Gaston 2002). This saturation effect implies that increases in R do not translate linearly into increases in FD. As a result, different FD metrics may converge in their responses, not necessarily because they capture similar ecological information, but because they are similarly constrained by the richness‐driven structure of the functional trait space. The residual‐based approach implemented here provides a simple yet effective way to control for this effect by isolating the variation explained by R prior to evaluating concordance. This procedure is not limited to the specific indices considered in this study but can be broadly applied to other FD metrics, regardless of whether richness is included explicitly in their formulation. Rather than assuming independence from R, our approach empirically tests whether the shared structure between metrics persists after accounting for its influence. In the present study, strong residual associations persisted for both wFDc–RQ and Q–FDis across the three ecosystems. The persistence of strong correlations in this context suggests that the agreement between indices reflects underlying structural concordance in the representation of FD. Building on this perspective, the saturation behaviour described above can be conceptually extended into what may be termed a functional saturation principle: once the occupied functional space is sufficiently represented, the addition of new taxa that do not expand the functional volume may produce negligible increases in metrics that primarily reflect the extent of occupied functional space. Although consistent with previously reported asymptotic FD–richness relationships (e.g., Mouillot et al. 2011), this principle has not been explicitly formalised as a standalone conceptual framework. Given the continuing lack of consensus regarding the definition and interpretation of functional traits and FD metrics (Bastos‐Pereira et al. 2022), this principle should be viewed as a conceptual guideline to help researchers understand how FD metrics scale and respond to richness constraints across ecosystems.

### 4.4 Implications for Modelling and Biomonitoring

Frequently, multiple FD indices are employed simultaneously under the assumption that they provide complementary information (e.g., Paz et al. 2023). However, the high level of concordance observed in this study suggests that different FD metrics may capture largely overlapping representations of functional structure, raising concerns about redundancy in ecological models. In ecological modelling, the simultaneous use of FD metrics that capture overlapping functional information may introduce collinearity, reducing model interpretability and compromising model parsimony (Dormann et al. 2013). Our framework provides a diagnostic tool for evaluating structural overlap between indices before their inclusion in statistical models. Identifying redundant indices allows researchers to adopt more parsimonious modelling strategies, enhancing both interpretability and robustness. From a biomonitoring perspective, index selection is often constrained by the need for methodological simplicity, reproducibility and scalability across large spatial and temporal datasets (Schleuter et al. 2010). Our results suggest that when multiple indices capture similar functional patterns, a single well‐performing metric may be sufficient. Indices such as RQ may offer practical advantages because of their relatively straightforward formulation and the absence of subjective decisions—such as the choice of clustering algorithms—required by dendrogram‐based approaches like wFDc (Petchey and Gaston 2007). Importantly, this does not imply that one index is universally superior; rather, the choice should be guided by both ecological relevance and statistical behaviour.

### 4.5 Limitations and Scope of the Framework

Although the results were consistent across analytical steps and datasets, several limitations should be acknowledged. First, the analyses were restricted to freshwater benthic macroinvertebrate communities identified at the family level, which may reduce functional variability and limit generalisation to communities with different taxonomic resolution or ecological complexity. Although the analytical structure of the framework is transferable, its performance should be evaluated across additional taxonomic groups, climatic regions and ecological systems. The influence of sample size on framework performance also deserves further evaluation. FD metrics may also be sensitive to sampling intensity and data structure, influencing their estimation and comparability across studies (van der Plas et al. 2017). Second, the framework depends on the definition and selection of functional traits. Because FD metrics are sensitive to the completeness and quality of trait information (Pakeman 2014), the validity of the results depends on the ecological relevance of the underlying trait data (Legras et al. 2019). The proposed framework evaluates statistical concordance and potential redundancy among selected FD metrics rather than their biological superiority. High statistical concordance should not be interpreted as ecological equivalence, as different indices may retain distinct heuristic value depending on the ecological context and analytical objective. The framework should therefore be viewed as a multi‐step diagnostic tool for evaluating selected FD metrics and identifying analytical redundancy before ecological modelling. Finally, the minimum input requirements of the FD Concordance R package and its companion Shiny application of the same name are computational safeguards, not guarantees of sufficient sample size; adequacy depends on model complexity, sampling design and the number of independent sampling units.

## 5 Conclusions

The two pairs of FD indices, wFDc versus RQ and Q versus FDis, exhibited consistently high concordance, which persisted after controlling for taxonomic richness. These patterns remained consistent across freshwater systems with differing ecological characteristics and moderately different sampling approaches, suggesting that the proposed framework is robust to moderate environmental and methodological variation. Across these empirical applications, the results show that selected FD metrics may produce highly similar patterns when there is an a priori rationale for expecting structural overlap. Rather than relying on a single threshold, redundancy was evaluated using complementary lines of evidence, including regression models, rank‐based associations, categorical agreement and residual concordance. Together, these analyses provided robust evidence of overlapping functional structure. The proposed framework offers a reproducible diagnostic approach for targeted comparisons between selected FD metrics and may support more parsimonious ecological modelling and clearer functional assessments in freshwater biomonitoring.

## Author Contributions

**Gonzalo Sotomayor:** conceptualization (lead), data curation (lead), formal analysis (lead), funding acquisition (lead), investigation (lead), methodology (lead), project administration (lead), resources (lead), software (lead), supervision (lead), validation (lead), visualization (lead), writing – original draft (lead), writing – review and editing (lead). **Marie Anne Eurie Forio:** conceptualization (supporting), data curation (supporting), investigation (supporting), resources (supporting), writing – review and editing (supporting). **Christine van der heyden:** conceptualization (supporting), data curation (supporting), investigation (supporting), resources (supporting), writing – review and editing (supporting). **Patricio X. Lozano Rodríguez:** conceptualization (supporting), data curation (supporting), funding acquisition (lead), investigation (supporting), resources (lead), supervision (lead), writing – review and editing (supporting). **Peter L. M. Goethals:** conceptualization (supporting), data curation (supporting), funding acquisition (lead), investigation (supporting), project administration (lead), resources (lead), supervision (lead), writing – review and editing (supporting).

## Conflicts of Interest

The authors declare no conflicts of interest.

## Supporting information


Supporting Information 1

## Data Availability

The Supplementary Materials provide the precomputed functional diversity index values used in the RQ–wFDc and Q–FDis comparisons for the three study ecosystems, together with taxonomic richness and sampling identifiers needed to reproduce the concordance analyses. These tables have also publicly available in Dryad https://doi.org/10.5061/dryad.6hdr7srhf. They support reproduction of the analyses from the calculated index values. The R package FD Concordance is available at https://github.com/Gonpa83/FD_concordance. Version 0.0.5 of the R package and companion Shiny source code is archived in Zenodo https://doi.org/10.5281/zenodo.23809787. The Shiny application can be accessed at https://01a168d6‐1a69‐a0e6‐7a29‐7e329e9dbbdb.share.connect.posit.cloud/. These tools enable researchers to apply the protocol to their own index values and generate reproducible figures and tables.
